# Variability of whole and peak match physical performance in highly trained female academy soccer players: A multi-club analysis

**DOI:** 10.1371/journal.pone.0318642

**Published:** 2025-02-12

**Authors:** Naomi Myhill, Dan Weaving, Nick Dalton Barron, Andy Hudson, Steve Barrett, Stacey Emmonds

**Affiliations:** 1 Carnegie School of Sport, Leeds Beckett University, Leeds, United Kingdom; 2 The Football Association, Burton Upon Trent, United Kingdom; 3 Prevent Biometrics, Minneapolis, Minnesota, United States of America; 4 Playermaker, London, United Kingdom; Università degli Studi di Milano: Universita degli Studi di Milano, ITALY

## Abstract

**Objective:**

Quantify between-match, -player and -team variability and compare whole- and peak-match locomotor characteristics between positions in elite female academy soccer.

**Method:**

Foot-mounted inertial measurement unit data were collected from 11 Women’s Super League Academy teams (n = 257 players; 171 matches). Differences between positions and variability were analysed using partial least squares correlation analysis (PLSCA) and linear mixed effects. Two latent variables were computed as composite scores of either whole match or peak intensity variables from the PLSCA.

**Results:**

Between-match variability of whole- and peak-match locomotor characteristics were similar (2 to 24% *vs* 0.2 to 22%). Between-team and -player variability was higher for whole- than peak-match locomotor characteristics (1 to 20% vs 0.1 to 3%, 8 to 112% *vs* 0.3 to 77%). From 30 pairwise comparisons, there were two *large* effect size (ES) differences (p < 0.001), WM had greater whole and peak match composite intensity than CDM. There were 10 *moderate* ES differences (p < 0.05), with WM greater than CD and CAM, F greater than CD and CDM and WD greater than CDM. All other comparisons were non-significant, *trivial* or *small*.

**Conclusion:**

Whole- and peak- match locomotor characteristics are similar across all outfield positions in elite female academy soccer. Between-match variability was greater for sprint distance than all other locomotor characteristics. Low variability between teams for peak locomotor characteristics means practitioners can be confident using peak reference values from this study and use them to evaluate training drill intensities of tactical-technical drills.

## Introduction

Progressing players from the youth to senior team is often a criterion for success for clubs and national governing bodies in soccer. Quantifying physical performance during matches can assist the development of youth players by identifying the determinants of performance in the sport and how their importance might differ across positions or levels of competition. Equally, understanding sources of variation in physical performance can be used to determine whether a change in physical performance can be considered normal or unusual to inform subsequent training prescription. However, despite the increase in professionalism and investment in the youth pathways in female soccer, there is currently a lack of research associated with high level female youth soccer players, particularly those close to the transition to first team football (e.g., U21).

Physical performance in soccer is a latent construct that is broadly conceptualised as either the amount (e.g., distance covered [m]) or the rate (e.g., average speed [m·min^−1^]) in which activity is completed. Multiple measurements are often used to represent the different sub dimensions of the physical performance construct (e.g., total distance, high speed running [HSR], acceleration, deceleration, sprinting) due to their different physiological effects. It is generally accepted that when representing the amount of activity, data collected across the whole match is used. For example, studies have reported whole match total distance (8202–9056 m), HSR (509–859 m) and sprint distance (SpD) (113–331 m) in U20 international matches [1] and whole match average speed (93 ± 21 m·min^−1^), HSR (3 ± 1.4 m·min^−1^) and sprinting (0.6 ± 1.4 m·min^−1^) in U16 domestic matches [2].

However, while an average rate across the whole match is useful for understanding average rates completed over a prolonged duration (i.e., 90 min), this data is likely less useful for evaluating tactical-technical training drill prescription, which is often much shorter duration (e.g., 4 min per set). Identifying the peak rates for varying durations using a moving average approach is accepted as a better method to support the understanding of the rates of physical performance and assist training prescription [3–8]. One study investigated the peak physical performance of U14 and U16 female soccer matches, reporting average speed 1 min: U14 = 156.6–165.6 m ⋅ min^−1^; U16 = 159.1–170.6 m ⋅ min^−1^; to 10-min: U14 = 103.5–118.1 m ⋅ min^−1^; U16 = 103.5–118.9 m ⋅ min^−1^ [2]. In senior female soccer, studies have recently explored peak match physical performance (average speed 1-min: 174 to 192 m ⋅ min^−1^; HSR 1-min: 71 to 93 m ⋅ min^−1^; SpD 1-min: 37 to 54 m ⋅ min^−1^; average speed 5-min: 127 to 142 m ⋅ min^−1^; HSR 5-min: 24.6 to 42 m ⋅ min^−1^; SpD 5-min: 11 to 18 m ⋅ min^−1^) [7,9,10]. Although the peak running speeds of senior female soccer and youth U14-U16 soccer have been quantified [2,7,9,10], a key omission is the academy age group (U21s), which is an important level to ensure players are exposed to appropriate running speeds (peak locomotor characteristics) to support preparing players to successfully progress to elite senior (Women’s Super League) soccer.

Player physical performances (e.g., HSR) are affected by contextual (e.g., ball possession and season phase) and internal factors (e.g., physiological capacity) which cause variation between matches [11]. Partitioning sources of variability such as between-match, between-player, within-player and in studies with multiple teams, between-team variability can help to determine whether a change in demands can be considered normal or unusual by establishing reference values for meaningful change. Previous research in female soccer has reported HSR and sprinting to have the most variability between matches (34 – 56%), with acceleration and deceleration having lower variability (0.3 – 17%) [7,9]. Although studies have reported sources of variability (between-match, between-player, between-team) in senior female soccer for whole match [7] and peak locomotor characteristics [9,12], most are either single team cohorts [7,12] or represent less than half of the teams in their respective league [9]. Therefore, findings may not be generalisable due to influences of individual club strategies, tactics or playing styles. Future research using multi-club data collection can overcome this. Furthermore, no study has reported sources of variability in elite academy female soccer. This is important as the magnitude of variability may be different in female academy than senior cohorts due to continual changes in physical development, which could lead to under- or overestimation of changes in performance and by proxy, internal load.

As such, there is a need for further research to quantify the peak locomotor characteristics of elite academy female soccer and sources of variability using a multi club approach. Therefore, the aims of the current study were to 1.) quantify the variability of whole- and peak-match physical performance of youth (U21) matches in England (WSL-A) and 2.) compare these physical performances between playing positions.

## Methods

### Experimental overview

An observational study design was conducted in which foot-mounted inertial measurement unit (IMU) data were collected during competitive league matches for 11 Women’s Super League Academy (WSL-A) clubs competing in the FA WSL Academy League (2021 to 2022 season). The amount (m) and rate (m ⋅ min^−1)^ of activity across the whole match for total-, high-speed- (HSR; 5.29 – 6.26 m ⋅ s^−1^) sprint- (SpD; > 6.26 m ⋅ s^−1^), acceleration- (>3 m ⋅⋅ s^−2^) and deceleration-distance (<-3 m ⋅ s^−2^) were quantified. Peak average running speeds, HSR, SpD, acceleration and deceleration (expressed as m ⋅ min^−1^) of specific positions were quantified using a moving average approach. Speed thresholds align with recent studies in female soccer [2,13–17], however there are currently no standardised velocity thresholds used in female soccer.

### Participants

Two hundred and fifty-seven players participated (age = 18 ± 1 years, mass = 62.6 ± 7.5 kg, height = 165.1 ± 12.2 cm) from 11 WSL-A teams across 171 competitive league matches. The study recruitment period ran from 01/05/2021 to 01/09/2021. Players were categorised into one of six outfield positions; central defender (CD; players per positional group [n] = 42), wide defender (WD; n = 52), central defensive midfielder (CDM; n = 18), central attacking midfielder (CAM; n = 71), wide midfielder (WM; n = 32) and forward (F; n = 41), aligning with previous research [12–14,16,18]. Goalkeepers were excluded from analysis (n = 22) and player observations of < 90 min duration for whole match and peak data were excluded (whole match [n = 1630], peak [n = 3178]), resulting in 1672 individual whole match observations and 3812 individual peak-match observations. This study was conducted according to the requirements of the Declaration of Helsinki and was approved by the university ethics committee of Leeds Beckett University (ref 73543). Parent or guardian consent was obtained by each club for players < 18 years.

### Procedures

Each player wore two 10 Hz foot-mounted IMUs (one for each foot; Playermaker, Tel Aviv, Israel) [13,14]. Concurrent agreement (mean difference: −0.05 ± 0.58 m ∙ s^−1^, root mean squared error: 0.58 m ∙ s^−1^) with 3D motion capture and between-device reliability (intraclass correlation: 0.84 *to* 1) has been reported [19]. All devices were activated via a Bluetooth connection to an iPad (Apple Inc, California) prior to each match. Data was uploaded to the manufacturer’s cloud-based software (v.3.22.0.02) post-match by club practitioners.

### Data analysis

Instantaneous speed data were exported from the manufacturer software and analysed in R Studio (v4.1.2; R Foundation for Statistical Computing, Vienna, Austria). Warm-up data were excluded. A custom-built script computed moving averages of speed (m ⋅ min^−1^), HSR (5.29 – 6.26 m ⋅ s^−1^; m ⋅ min^−1^), sprinting (> 6.26 m ⋅ s^−1^; m ⋅ min^−1^), average acceleration (m ⋅ min^−1^) and average deceleration (m ⋅ min^−1^), over 1–10 min. The maximum value for each player and duration per match was determined. 1-, 3- and 5-min were selected for statistical analysis to represent common drill prescription durations. The power-law relationship was calculated for each variable (average speed, HSR, SpD, acceleration and deceleration) by calculating the intercept and slope using the 1 to 10-min peak durations (Delaney, Thornton (4)).

### Statistical analysis

Descriptive data are presented as mean ± SD. To estimate sources of variability (between-team, between-player, between-match, within-player), log transformed dependent variables of each individual whole match or peak intensity variable were modelled using linear mixed effects (*lme4* package in R Studio [version 4.1.2]). Fixed effects of position (CD, WD, CDM, CAM, WM, F) and random effects of player-, team- and match-identity were specified. Model assumptions of normality and homoscedasticity of residuals, multicollinearity, and autocorrelation were assessed, and were unviolated. Standard deviation of each random effect was expressed in percentage units (coefficient of variation % [CV%]) by back-transforming each estimate.

To compare positional groups, minimise pairwise comparisons, a twostep modelling approach was taken. First, partial least squares correlation analysis (PLSCA) models were produced as per previous methods [20,21]. Data (either whole match or peak intensity variables) were mean centred and standardised and divided into two matrices, *X* containing either whole match or peak variables for each player observation and *Y* containing binary variables to represent position (CD, WD, CDM, CAM, WM, F). Saliences (weights) for matrix *X* from PLSCA were produced for the 1^st^ dimension for each set of variables. These saliences were multiplied by each player’s original mean centred and standardised data to provide a single composite value for each player and match observation [22]. The saliences (weights) are the linear weighted contribution of the original variables for *X* (e.g., whole match or peak match) to each dimension of the PLSCA model. By multiplying the saliences with the original mean centred and standardised data for each original variable – a latent variable score can be created for each observation. Therefore, the latent variables are a composite score of either whole match or peak intensity variables from the PLSCA. Secondly, this latent variable was inputted as a dependent variable into a mixed effect model with the same model structure (fixed and random effects). This twostep process was repeated for both whole match and peak intensity variables. Tukey’s pairwise comparisons between positions were conducted using the least squares mean test (*lmerTest* package). Cohen’s *d* effect size (ES) statistics were calculated (ratio of pooled SDs) with 90% confidence intervals (CI). ES was classified as *trivial* (< 0.2), *small* (0.2 to 0.59), *moderate* (0.6 to 1.19), *large* (1.2 to 2), *very large* (> 2) [23].

## Results

### Variability of whole match and peak match locomotor characteristics

The variability of peak and whole match physical performances are presented in Tables 1 and 2 respectively. CV values of whole match physical performance ranged from 1% to 112%, with the lowest CVs associated with between-team variability for acceleration distance per min (1%) and the highest with between-player variability of SpD (112%). All sources of variability for whole match variables were greater for SpD (20–112%) than all other variables. Between-player and within-player variation was higher in the whole match variables, except average speed, than other sources of variation. CV values for 1-, 3- and 5-min peak variables ranged from 0% to 95%, with the lowest CVs associated with between-team variability for peak 1-, 3- and 5- min acceleration (0%), and the highest with within-player variability of peak 1-, 3- and 5- min SpD (56% – 95%).

**Table 1:** Sources of variability for peak locomotor characteristics in academy match play.

	Epoch length	Between-match CV%	Between-team CV%	Between-player CV%	Within-player CV%
Average speed	1	5%	1%	5%	27%
	3	6%	3%	6%	30%
	5	7%	4%	6%	31%
HSR	1	17%	3%	29%	54%
	3	16%	2%	31%	47%
	5	16%	0%	33%	44%
SpD	1	22%	3%	77%	95%
	3	16%	1%	55%	67%
	5	14%	0%	48%	56%
Acceleration	1	10%	0%	23%	35%
	3	6%	0%	15%	22%
	5	5%	0%	13%	18%
Deceleration	1	10%	5%	16%	32%
	3	7%	4%	14%	24%
	5	6%	3%	13%	20%

**Table 2:** Sources of variability of whole match characteristics for academy match play.

	Between-match CV%	Between-team CV%	Between-player CV%	Within-player CV%
Distance covered	3%	6%	26%	37%
Average speed	11%	2%	8%	26%
HSR covered	16%	16%	63%	56%
HSR per min	14%	6%	30%	27%
Sprint distance	24%	20%	112%	99%
Sprint distance per min	6%	4%	23%	18%
Acceleration distance (>3 m ⋅ s^−2^)	16%	3%	52%	54%
Acceleration distance per min	2%	1%	6%	6%
Deceleration distance (<-3 m ⋅ s^−2^)	17%	12%	48%	51%
Deceleration distance per min	3%	2%	9%	9%

### Whole match locomotor characteristics

**Fig 1:**
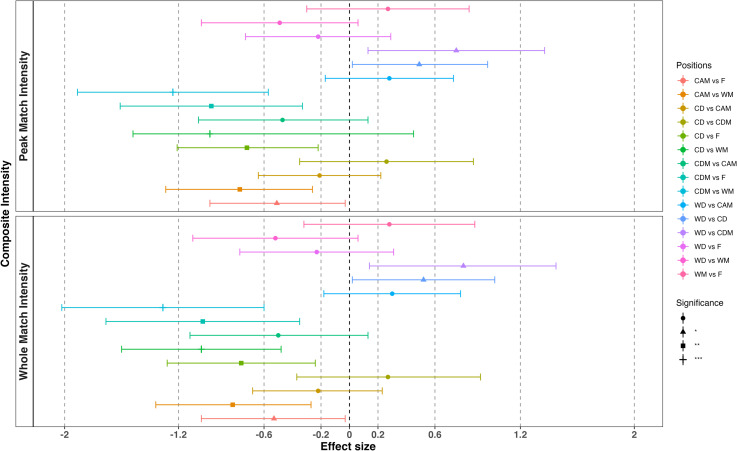
Effect size differences for whole match and peak match composite intensity by position.

The average whole match physical performance variables for each position are presented in Table 3. There was no overall difference between positions in whole match intensity for WSL-A matches. Fig 1 presents the ES differences between positions for the composite whole match intensity score. From this there was one significant (p < 0.001) *large* ES difference with a greater whole match intensity for WM than CDM, and five significant (p < 0.05) *moderate* ES differences, with WM greater than CD and CAM, F greater than CD and CDM and WD greater than CDM. All other comparisons were non-significant, *trivial* or *small*. S1 Table reports the individual variable contribution (saliences [weights]) to the construction of the latent whole match intensity variable from PLSCA (1^st^ dimension).

**Table 3:** Average whole match running characteristics (mean ± SD).

	Overall	CD	WD	CDM	CAM	WM	F
Total distance (m)	8940 ± 1094	8529 ± 846	9016 ± 1048	9003 ± 1139	9258 ± 1179	9183 ± 992	8644 ± 1192
Average speed (m ⋅ min^−1^)	92 ± 11	88 ± 9	93 ± 11	93 ± 11	96 ± 13	95 ± 11	90 ± 12
HSR (m; > 5.29 m ⋅ s^−1^)	262 ± 137	225 ± 109	294 ± 130	210 ± 110	219 ± 130	352 ± 145	324 ± 143
HSR per min (m ⋅ min^−1^)	3 ± 1	2 ± 1	3 ± 1	2 ± 1	2 ± 1	4 ± 2	3 ± 1
SpD (m; > 6.26 m ⋅ s^−1^)	39 ± 39	36 ± 34	41 ± 38	31 ± 33	26 ± 33	59 ± 44	57 ± 54
SpD per min (m ⋅ min^−1^)	0.4 ± 0.4	0.4 ± 0.3	0.4 ± 0.4	0.3 ± 0.3	0.3 ± 0.3	0.6 ± 0.5	0.6 ± 0.6
Acceleration Distance (m; > 3 m ⋅ s^−2^)	12 ± 8	11 ± 7	12 ± 8	10 ± 6	10 ± 8	16 ± 10	14 ± 9
Acceleration per min (m ⋅ min^−1^; > 3 m ⋅ s^−2^)	0.12 ± 0.08	0.12 ± 0.07	0.12 ± 0.08	0.10 ± 0.06	0.11 ± 0.08	0.17 ± 0.10	0.15 ± 0.09
Deceleration Distance (m; < -3 m ⋅ s^−2^)	28 ± 15	22 ± 13	30 ± 16	23 ± 13	26 ± 14	35 ± 16	35 ± 17
Deceleration per min (m ⋅ min^−1^; < -3 m ⋅ s^−2^)	0.29 ± 0.16	0.23 ± 0.13	0.31 ± 0.17	0.23 ± 0.13	0.27 ± 0.14	0.36 ± 0.16	0.36 ± 0.18

### Peak match characteristics

**Fig 2:**
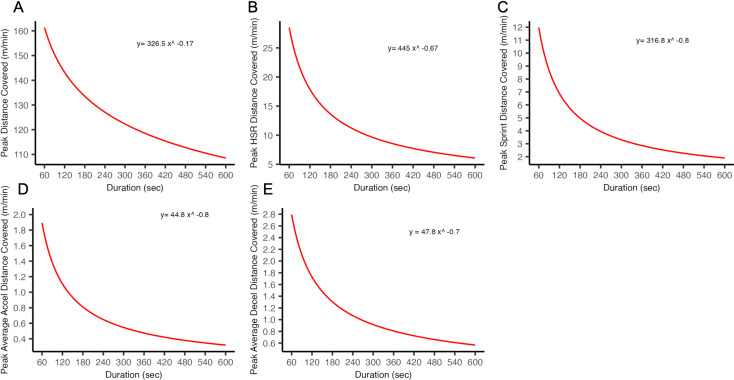
Power Law relationship for maximal duration-specific A) Average speed, B) High Speed Running, C) Sprinting, D) Acceleration and E) Deceleration.

The average 1-, 3- and 5-min peak physical performances per position are presented in Table 4. Intercepts and slopes and power-law relationship for peak physical performances are presented in Table 5 and Fig 2.

**Table 4:** Peak 1-, 3-, 5-min values for each position for full match players (Mean ± SD).

Locomotor characteristic	Epoch length	Overall *(n = 3812)*	CD *(n = 844)*	WD *(n = 716)*	CDM *(n = 338)*	CAM *(n = 860)*	WM *(n = 560)*	F *(n = 494)*
Average Speed (m ⋅ min^−1^)	1	166 ± 20	159 ± 15	168 ± 20	163 ± 21	170 ± 21	170 ± 23	164 ± 22
	3	131 ± 17	125 13±^d^^♦^	132 ± 17	130 ± 19	136 ± 18^a^^♦^	133 ± 19	129 ± 18
	5	121 ± 17	116 ± 12	122 ± 16	121 ± 19	125 ± 17	123 ± 18	120 ± 17
High Speed Running (m ⋅ min^−1^)	1	30 ± 14	27 ± 12	32 ± 13	26 ± 13	29 ± 14	36 ± 15	31 ± 14
	3	13 ± 6	11 ± 5	14 ± 6^c * ^	11 ± 6^e^^♦^	12 ± 7^e * ^	16 ± 7^c^^♦^	14 ± 7^c * ^
	5	9 ± 5	8 ± 4^e • ^	10 ± 5	7 ± 4^ef^^♦^	9 ± 5^e^^♦^	12 ± 5 cd^♦^	10 ± 5^c^^♦^
Sprint Distance (m ⋅ min^−1^)	1	10 ± 10	9 ± 9	11 ± 9	8 ± 9	9 ± 10	16 ± 11	11 ± 12
	3	4 ± 4	3 ± 3	4 ± 3	3 ± 3	3 ± 4	6 ± 4	4 ± 5
	5	2 ± 3	2 ± 3	3 ± 2	2 ± 2	2 ± 3	4 ± 3	3 ± 3
Average Acceleration (m ⋅ min^−1^)	1	2 ± 1.1	1.9 ± 1	2 ± 1	1.6 ± 0.9	1.9 ± 1.3	2.4 ± 1.2	2.1 ± 1.2
	3	0.8 ± 0.5	0.7 ± 0.4	0.8 ± 0.5	0.6 ± 0.4	0.7 ± 0.6	1 ± 0.6	0.9 ± 0.6
	5	0.5 ± 0.4	0.5 ± 0.3^e * ^	0.5 ± 0.3	0.4 ± 0.3^e^^♦^	0.5 ± 0.4	0.7 ± 0.4	0.6 ± 0.4^c * ^
Average Deceleration (m ⋅ min^−1^)	1	3 ± 1.3	2.7 ± 1.3	3.1 ± 1.3	2.4 ± 1.1	2.8 ± 1.3	3.4 ± 1.4	3.2 ± 1.4
	3	1.3 ± 0.7	1.1 ± 0.6^ef^^♦^	1.3 ± 0.6 cd^♦^	1 ± 0.5^ef^^♦^	1.2 ± 0.7^cf * ^	1.5 ± 0.7^d * ^	1.4 ± 0.8
	5	0.9 ± 0.5	0.8 ± 0.4^ef^^♦^	1 ± 0.5^c^^♦^	0.7 ± 0.4^ef^^♦^	0.9 ± 0.5^ce * ^	1.1 ± 0.6	1 ± 0.5

N.B. Significantly different to ‘a’ CD, ‘b’ WD, ‘c’ CDM, ‘d’ CAM, ‘e’ WM, ‘f’ F. Significance level 0.05 ‘•’, 0.01 ‘*’, 0.001 ‘♦*’*

**Table 5:** Intercept and slopes from the power-law relationship for estimating match intensity by duration for academy female soccer players.

Locomotor characteristic		Overall
Average speed	Intercept	326
	Slope	−0.17
HSR	Intercept	445
	Slope	−0.67
Sprint Distance	Intercept	316.7
	Slope	−0.85
Acceleration Distance	Intercept	45
	Slope	−0.77
Deceleration Distance	Intercept	48
	Slope	−0.69

Fig 1 presents the ES differences between positions for the composite peak match intensity. There is no overall difference between positions in peak match intensity for WSL-A matches. There was one significant (p < 0.001) *large* ES difference with greater peak match intensity for WM than CDM and five significant (p < 0.05) *moderate* ES differences with WM greater than CD and CAM, F greater than CD and CDM and WD greater than CDM. All other comparisons were non-significant, *trivial* or *small*. S1 Table highlights the latent variable (peak-match composite intensity) created from the partial least squares model showing the saliences (weights) of each of the external intensity measures (1^st^ dimension).

## Discussion

This study aimed to quantify the variability of whole match and peak match physical performance variables in WSL-A matches and compare these between positions using a multi-club analysis. The between-match and within-player variability of whole match and peak match physical performances were similar (between-match: 2 to 24% *vs* 5 to 22%; within-player: 6 to 99% *vs* 18 to 95%), whereas between-team and between-player variability was higher for whole match than peak variables. However, all sources of variability were greatest for SpD than for other locomotor characteristics in whole match and peak 1-, 3-, 5-min durations, except for between-team variability for peak 1-, 3- and 5-min deceleration. Overall, there was a lack of substantial differences between positions for whole match and peak match intensity.

The between-match variability of whole match and peak match physical performance in the current study were similar, which contrasts with the higher variability for peak 1-min than whole match for TD (6.5% vs 4.6%), HSR (18.7% vs 15.9%) and acceleration and deceleration (12.9% vs 11.7%) reported in elite senior female soccer [9]. This is an interesting finding as peak physical performances have been suggested to be more variable than whole match [24], and while the current findings report there is variability (5% to 22%), the similarity between peak and whole match suggests that the variability is intrinsic to any match-to-match analysis approach. Variability (within-player and between-match, team and player) appears to be higher in the current study (0% to 99%) than previous analysis of an elite senior female soccer cohort (0.3% to 37.2%) [9]. This could be explained by a larger player and team sample size in the current study (11 out of a total of 14 WSL-A teams) which has likely increased data heterogeneity and reduced the risk of bias caused by a specific style of play [25]. Furthermore, the speed thresholds used in the current study are higher (HSR: 5.29 – 6.26 m ⋅ s^−1^ *vs* 4.44 – 5.55 m ⋅ s^−1^; SpD > 6.26 m ⋅ s^−1^ *vs* >  5.55 m ⋅ s^−1^) and variability tends to increase with running speed [26]. However, between-match variability for peak 1-, 3- and 5- min physical performance in the current study (CV <  22%) is lower than recent research in senior female soccer (CV >  28.5%; [12]) and another study reports greater peak 5-min HSR variability (31%) than the current study (16%). This may be explained by the fact the authors used lower speed thresholds (HSR: > 4.58 m ⋅ s^−1^, SpD: >  5.55 m ⋅ s^−1^) [7]. Between match, between-player and within-player variability are greater for SpD than for other variables across both whole match and peak approaches, similar to previous research in female and male soccer [9,11,26], as variability tends to increase with running speed [26]. This suggests that exposures to TD, HSR, SpD volume and intensity from match-to-match will be inconsistent. There was little variability between teams for peak 1-, 3-, 5- min variables (CV < 5%), this means academy practitioners and coaches can be confident in using the presented reference values for their team. However, the variability between teams for whole match variables were higher (CV < 21%), suggesting that the differences in physical performance between teams may be captured across 90-min. This is likely due to the interaction with tactical instruction and stoppages in play, as previously reported [8,27].

Generally, the specific positional differences (i.e., *large* and *moderate* ES differences), show WM perform higher intensities than CDM across whole match and peak match composite intensities. However, the wide confidence intervals for all positional comparisons suggest uncertainty in the estimate. As such, the findings suggest that overall, there are no positional differences in the whole match and peak locomotor match characteristics of elite female academy soccer players. This contrasts with previous research in senior female soccer which reported lower whole match locomotor characteristics for central players than other outfield positions [10,18,28–30] and the same for peak locomotor characteristics [10,12]. For example, Winther, Baptista (10) reported significant differences in SpDsp between wide players (whole match: WD = 413 ± 53, WM = 530 ± 59 m, peak 1-min: WD = 53 ± 4, WM = 54 ± 5 m) and central players (whole match: CD = 227 ± 54, centre midfield = 293 ± 47 m, peak 1-min: CD = 37 ± 4, centre midfield: 40 ± 4 m), the difference in peak 1-min SpD between WM and centre midfield is 0.4 m ⋅ s^−1^ however the ES differences and CIs between position were not reported in the study therefore it is difficult to determine the certainty of the estimate. Furthermore, methods need to be developed to be able to assess the physical match characteristics for fluid positional transitions in soccer in attack and defence, which is an evolving tactical element of current match play. A major finding of this study indicates that WSL-A outfield players have similar physical performances during a match, regardless of their position. Coaches could use this information to support tactical and player development decisions.

Overall TD was 8831 ± 1398 m, which is similar to previously reported in international U20 match play (8674.9 ± 663.1 m) [31]. TD was slightly lower than (9274 – 10,572 m [28], 9275 – 10,797 m [29], 10,025 ± 775 m [30]) and similar (8934 – 10,131 m [10]) to previously reported in senior female soccer. Overall the largest differences between academy and senior female soccer are found in HSR (271 ± 156 m) and SpD (43 ± 50 m), which are lower in this study than previously reported in elite senior female soccer using similar speed thresholds (HSR [5.27 – 6.38 m ⋅ s^−1^] = 215 – 445 m; SpD [ > 6.38 m ⋅ s^−1^] = 45 – 136 m) [29], and identical speed thresholds (HSR = 316 – 585 m; SpD = 59 – 187 m) [16]. This finding is supported by previous studies which suggest that the amount of high intensity running, e.g., HSR or SpD, increases with competition level [13,32–34] and HSR has been shown to be closely related to fitness and training status [35]. Preparation for the most intense periods of match play is critical as these periods are often linked to key moments such as goal scoring [36]. Our findings indicate that the most demanding overall external intensities for WSL-A players during matches were 166 ± 20 m ⋅ min^−1^, 30 ± 14 m ⋅ min^−1^, 10 ± 10 m ⋅ min^−1^, 2 ± 1.1 m ⋅ min^−1^, 3 ± 1.3 m ⋅ min^−1^, for average speed, HSR, SpD, acceleration and deceleration distance respectively. While recent studies have started to quantify the peak locomotor characteristics in senior female soccer [7,10,12], it is difficult to compare peak- HSR, SpD, acceleration and deceleration distance due to the varied speed and acceleration thresholds used. Yet, our study reports similar peak 1-, 3- and 5-min average speeds (166 ± 20, 131 ± 17, 121 ± 17 m ⋅ min^−1^) in academy players to that reported in senior female soccer players in the Spanish 1^st^ league (168 ± 16, 133 ± 13, 124 ± 12 m ⋅ min^−1^) [12]. Conversely, peak 5-min average speed for WSL-A players (121 ± 17 m ⋅ min^−1^) is lower than previously reported for a senior international cohort (141 ± 12 m ⋅ min^−1^) [7]. When compared to U14 and U16 elite female soccer players (average speed peak 1-min: U14 = 156.6–165.6 m ⋅ min^−1^; U16 = 159.1–170.6 m ⋅ min^−1^; HSR peak 1-min: U14 = 28.6 – 34.4 m ⋅ min^−1^; U16 = 28.6 – 42.6 m ⋅ min^−1^) [2], the peak 1-min average speed and HSR observed in the current study are similar. However, match duration was less than the current study (70 min for U14 and 80 min for U16), which is likely to affect intensity of the matches. Despite a higher HSR threshold (5.29 – 6.26 m ⋅ s^−1^) the reported peak 1-min HSR distance in the current study (30 ± 14 m ⋅ min^−1^) is similar to previously reported in elite senior female soccer using a lower HSR speed threshold (>5 m ⋅ s^−1^; peak 1-min HSR = 30 m ⋅ min^−1^) [12]. Peak 1-min locomotor characteristics appear far greater than the relative mean values from the whole match. It is possible that for HSR and SpD measures, 1 min periods are likely to be completed within a single effort and shorter durations might better capture this [37].

### Practical applications

Practitioners can use the variability findings (Tables 1 and 2) to identify meaningful changes in the amount (i.e., volume) and rate (i.e., intensity) of activity between matches. For instance, a difference of 18% in whole-match HSR from the previous match would indicate a move to a meaningful change in HSR volume. This can inform training programming. The power-law relationship of peak physical performances reported in the study can act as reference values to evaluate tactical-technical drill intensity in female youth academy players. For example, when prescribing a 270 sec (4.5 min) tactical-technical drill aimed at replicating the peak average speed of WSL-A matches, using the intercept and slope values in Table 5, match intensity can be calculated as a function of time, where (*t* ) is drill time in seconds and (*i*) is peak intensity:


$$i=326t^{-0.17}$$ (1)

$$i=326x270^{-0.17}$$

i=126

This results in an estimated target speed of ~ 126 m ⋅ min^−1^ for the 4.5-min drill, which can then be compared to an individual player’s output during training. These calculations can be integrated into a team’s monitoring system, to allow the intensity of tactical-technical drills to be assessed over time and enabling coaches to periodise exposure to peak match physical performances when appropriate.

## Conclusion

Between-match variability was greatest for sprint distance than all other physical performance variables. Whole match and peak match locomotor characteristics are similar across all outfield positions in elite academy female soccer. There was little variability between teams for peak physical performance variables so academy practitioners can be confident using the peak reference values from this study to evaluate tactical-technical drill intensities. Findings and reference values can provide coaches and practitioners with insight into the physical performances of elite youth female soccer players and their variation.

## Supporting Information

S1 Table Construction of whole-match and peak-match latent external intensity variables from the partial least squares correlation model. N.B. Values represent the saliences (weights; 1^st^ dimension).
(DOCX)
