# Supplementary material for: Variability of whole and peak match physical performance in highly trained female academy soccer players: A multi-club analysis
Source: PLoS One. 2025 Feb 12;20(2):e0318642. doi: 10.1371/journal.pone.0318642 (PMC11819598; doi:10.1371/journal.pone.0318642)
Supplement: S1 Table — N.B. Values represent the saliences (weights; 1st dimension). (DOCX) [file pone.0318642.s001.docx]

**S1 Table.** **Construction of whole-match and peak-match latent external intensity variables from the partial least squares correlation model.**

| Latent external intensity variable | Locomotor characteristic | Saliences |
| --- | --- | --- |
| Whole-match external intensity | Total distance (m) | 0.17 |
|  | Average speed (m×min^-1^) | 0.18 |
|  | HSR (m) | 0.41 |
|  | HSR per min (m×min^-1^) | 0.41 |
|  | SpD (m) | 0.29 |
|  | SpD per min (m×min^-1^) | 0.29 |
|  | Acceleration Distance (m) | 0.29 |
|  | Acceleration per min (m×min^-1^) | 0.29 |
|  | Deceleration Distance (m) | 0.37 |
|  | Deceleration per min (m×min^-1^) | 0.36 |
| Peak-match external intensity | Peak 1-min speed (m×min^-1^) | 0.19 |
|  | Peak 1-min HSR (m×min^-1^) | 0.25 |
|  | Peak 1-min sprint (m×min^-1^) | 0.21 |
|  | Peak 1-min acceleration (m×min^-1^) | 0.25 |
|  | Peak 1-min deceleration (m×min^-1^) | 0.26 |
|  | Peak 3-min speed (m×min^-1^) | 0.17 |
|  | Peak 3-min HSR (m×min^-1^) | 0.32 |
|  | Peak 3-min sprint (m×min^-1^) | 0.22 |
|  | Peak 3-min acceleration (m×min^-1^) | 0.26 |
|  | Peak 3-min deceleration (m×min^-1^) | 0.32 |
|  | Peak 5-min speed (m×min^-1^) | 0.15 |
|  | Peak 5-min HSR (m×min^-1^) | 0.35 |
|  | Peak 5-min sprint (m×min^-1^) | 0.24 |
|  | Peak 5-min acceleration (m×min^-1^) | 0.27 |
|  | Peak 5-min deceleration (m×min^-1^) | 0.31 |

*N.B. Values represent the saliences (weights; 1^st^ dimension).*
